# Isolation, characterization and genome analysis of an orphan phage FoX4 of the new *Foxquatrovirus* genus

**DOI:** 10.1186/s12866-022-02719-3

**Published:** 2022-12-13

**Authors:** D. Holtappels, K. J. Fortuna, M. Vallino, R. Lavigne, J. Wagemans

**Affiliations:** 1 Department of Biosystems, KU Leuven, Leuven, Belgium; 2 Institute of Sustainable Plant Protection, National Research Council of Italy, Turin, Italy

**Keywords:** Bacteriophage, Genomics, *Xanthomonas campestris*, Phage biocontrol

## Abstract

The growing interest in the therapeutic application of bacteriophages leads to a drastic increase in the number of sequenced genomes. Luckily, recent insights in phage taxonomy facilitate the classification of phages in a comprehensive and data-driven manner as recently proposed by the International Committee on Taxonomy of Viruses. In this research, we present the taxonomical classification of a novel, narrow host range *Xanthomonas* phage FoX4, isolated from a Brussels sprouts field in Belgium infested with *Xanthomonas campestris* pv. *campestris*. The phage has a limited ability to lyse a bacterial culture, yet adsorbs efficiently to its host. Based on its genome sequence and low similarity to previously described phages, the phage comprises the novel phage genus *Foxquatrovirus*.

**Supplementary Information:**

The online version contains supplementary material available at 10.1186/s12866-022-02719-3.

## Introduction

Viruses are considered the most abundant entities in the world with an estimated 10^31^ particles, including bacteriophages, i.e. viruses infecting bacterial hosts [1]. An important group of bacterial viruses belong to the class of the *Caudoviricetes*, the tailed bacteriophages. Within this group, three virion morphologies can be distinguished: myoviruses with contractile tails, podoviruses with a short tail structure and siphoviruses with long non-contractile tails [2]. Traditionally, these morphotypes were translated into three phage families. Recent sequencing technologies, however, have revolutionized our understanding of the complexity and diversity of these viruses. New insights in the taxonomy of the tailed phages were gathered and showed that the complexity reached far beyond the diversity based on particle morphology. As such, major shifts in phage taxonomy have recently been proposed by the International Committee on Taxonomy of Viruses (ICTV) suggesting a.o. the abolishment of the traditional *Myoviridae* *,* *Podoviridae* and *Siphoviridae* families and raising subfamilies to the level of families [3]. This creates a big opportunity in the genomics of bacteriophages and the reorganization of unclassified viruses.

According to a review conducted by Zrelovs and colleagues, the total number of complete phage genomes counted over 7,718 in 2020. This number is an underrepresentation of the current figures, especially since the interest in phages as therapy or biocontrol strategies in different fields such as human medicine and agriculture is increasing [4]. More than half of the total complete genomes were previously classified as *Siphoviridae* [4]. However, phages with the distinct siphovirus morphology are also found in other families such as the *Demerecviridae* and *Drexlerviridae*, further supporting the abolishment of the morphotype-based family distinction in phage taxonomy. As a result, the classification of many siphoviruses is now incomplete.

The overall growing interest in phage biocontrol is especially true for crop protection. Lately, the number of phages reported that infect plant pathogenic bacteria has increased drastically [5–7]. Multiple bacterial genera and species are targeted and crop specific application strategies are developed. One particular bacterial species of interest is *Xanthomonas campestris* pv. *campestris* (Xcc)*.* This bacterium is the causal agent of black rot disease in brassica crops and arugula [8, 9]. Xcc is a Gram-negative bacterium that is known to grow epiphytically on leaves of crucifers and infects the plant through hydathodes or wounds. Infected weeds and crop residues have been described to function as a reservoir from where the bacteria can spread [8, 10–12]. Back in 1924, the first field trials with phages were conducted to control black rot disease [13]. In more recent years, several authors have revived the research on the development of a phage-based biocontrol strategy. Nagai and colleagues described for example the application of an Xcc phage pXcpSFC211 (pXS) together with a non-pathogenic *Xanthomonas* strain reducing disease severity between 2.7% and 18.9% compared to 43.3% and 93.7% in the controls. They further showed symptoms reductions by 24% in field conditions [14]. Similarly, our group published the potential of two Xcc phages in seed decontamination, bioassays in greenhouse conditions and field trials [5]. Papaianni and colleagues did not only show the potential of their Xcc phage as a biocontrol agent, but also showed its synergy with hydroxyapatite and long fatty acids to reduce Xcc biofilms [15, 16]. They further showed that cabbages do react on the presence of phage by themselves as they demonstrated decreased levels of amino acids- and nitrogen-containing compounds. Additionally, they demonstrated that in the presence of both the phage and the bacterium, significant differences are observed in the metabolic profiles, suggesting a response of the plant in the presence of phage [17]. Combined, these results illustrate the potential of bacteriophages as a valid biocontrol strategy.

In this research, we characterized novel *Xanthomonas* phage FoX4. We investigated the host range of the phage using an extensive bacterial collection isolated in Flanders (Belgium) along with the infection characteristics of the phage. Furthermore, based on whole genome sequencing, we analyzed the phylogeny of the phage and confirmed the annotation of the structural cassette using a gel-free mass spectrometry analysis.

## Materials and methods

### Phage isolation and transmission electron micrographs

FoX4 was isolated as described elsewhere [5]. In short, Xcc strain GBBC 1484 was grown to an optical density (600 nm; OD600) of 0.3 in 4 mL tubes. Xcc infested soil collected from infested fields was added to the growing bacterial culture (0.5 g). The mixture was incubated overnight at 25 °C, centrifuged (4,000 rpm, 10 min, 4 °C), filtered using 0.45 µm filters and spotted on a bacterial lawn of GBBC 1484. Lysis zones were picked with sterile toothpicks and plated using the double agar technique. Single plaques were picked up three consecutive times to obtain a monoclonal phage stock. Phages were propagated as previously described [18]. Transmission electron micrographs were made as described by Martino and colleagues [19].

### Characterizing the phage infection

The host range of FoX4 was determined by assessing its ability to form plaques on a particular strain. We used our previously reported strain collection [5]. Phages were spotted (3 µL) in a dilution series (10^6^ – 10^5^ – 10^4^ PFU/mL) on a bacterial lawn (200 µL of overnight culture in 4 mL of soft agar) of a specific strain and incubated overnight at 25 °C. Strains were considered susceptible to the phage if individual plaques were observed. The experiment was conducted in duplicate and strains that showed plaques in both assays were considered susceptible to the phage.

The lysis activity of the phage in liquid broth was asses by measuring the optical density of an exponentially growing bacterial culture (GBBC1412 at OD_600_ of 0.3) infected with phage at different multiplicities of infection (MOIs) (0.1 – 1) for nine hours. The adsorption rate of FoX4 was determined as previously described [20].

### Genome sequencing, annotation and proteome analysis

Phage DNA was isolated from the virions as described by Sambrook and Russel (2001). In short, polyethylene glycol (PEG8000)-precipitated phage stocks were treated with DNase I and RNase A for 30 min at 37 °C. Next, 10% SDS, 50 mM EDTA and proteinase K were added and incubated for one hour at 56 °C to break down the virions. A phenol/chloroform extraction followed by an ethanol precipitation was performed to clean up the phage DNA. The phage DNA was sequenced on an Illumina MiSeq platform (VIB Nucleomics Core, Belgium) and analyzed as previously described [5]. In short, reads were trimmed with Trimmomatic v0.39 and assembled using Unicycler v0.5.0 [21, 22]. The quality of the assembly was visualized by Bandage v0.9.0 [23]. Phage genomes were automatically annotated using the RASTtk pipeline [24] and manually curated. By means of primer walking, the physical ends of the genome were determined. Phage structural proteins were extracted by methanol-chloroform extraction as described previously, followed by in-gel trypsinization [25, 26]. Eluted peptide mixtures were analyzed with liquid chromatography tandem mass spectrometry (LC–MS/MS) on an Ultimate 3000 RSLC nano-LC (Thermo Fisher Scientific, Bremen, Germany) in-line coupled to a Q Exactive mass spectrometer (Thermo Fisher Scientific) at the VIB Proteomics Core (Belgium). Peptides were identified using the MaxQuant algorithm (version 2.0.2.0). Spectra were searched against the annotated phage proteome.

## Results

### Isolation and microbiological characterization of *Xanthomonas* phage FoX4

**Fig. 1:**
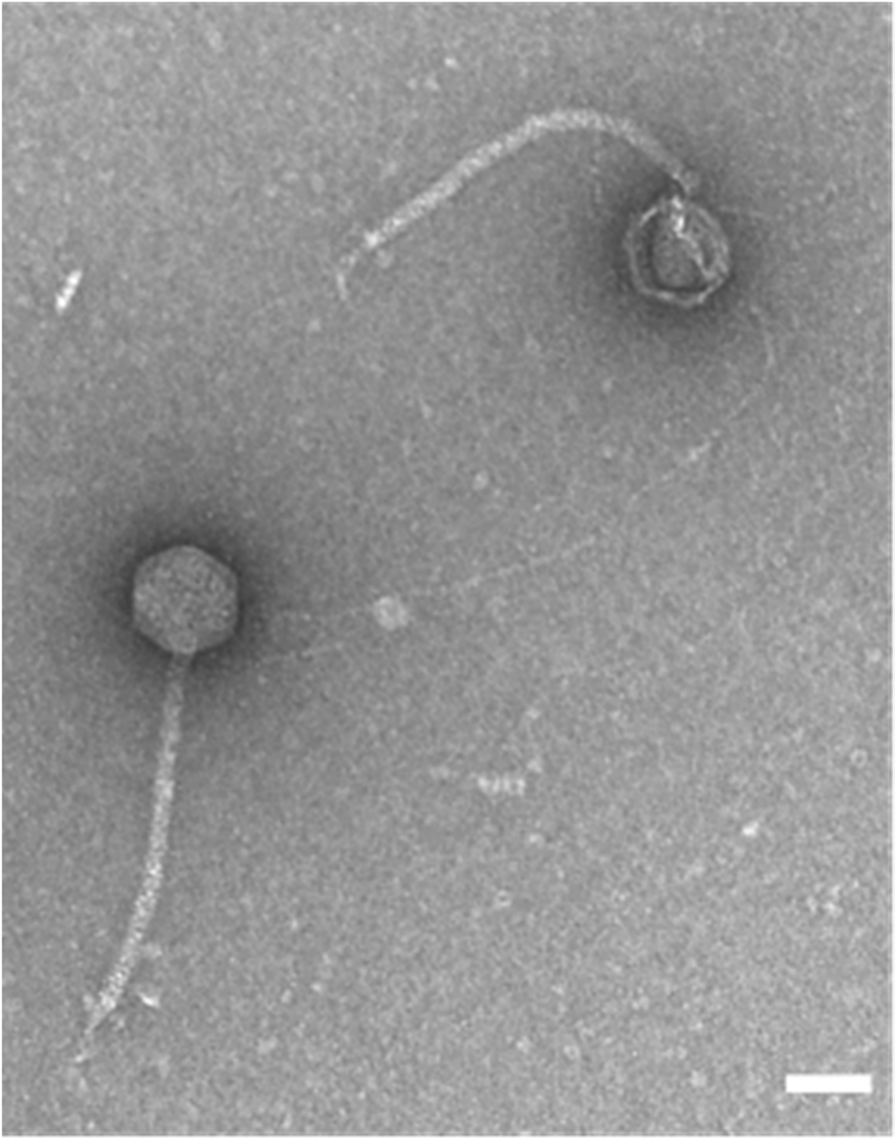
Transmission electron micrograph of FoX4 showing a typical siphovirus morphology. Scale bar represents 50 nm

Soil collected from the roots of symptomatic Brussels sprouts was incubated with Xcc GBBC 1484, the strain isolated from the same sample. Small turbid plaques were obtained with a diameter of approximately 1 mm. Based on transmission electron micrographs, FoX4 has a typical siphovirus morphology (Fig. 1).

The phage had a relatively narrow host range and infects 27% of our collection of 69 diverse strains (Supplementary Table 1). There appeared to be no relation between the susceptibility of a strain and the crop from which it was isolated. Despite its narrow host range, FoX4 could infect one of the *Xanthomonas campestris* pv. *raphani* (Xcr) strains in collection, GBBC 1468, suggesting shared phage susceptibility determinants between Xcc and Xcr for this phage. Furthermore, FoX4 infected the historical reference strains LMG 8052 and LMG 566, isolated in 1986 and 1941, respectively.

**Fig. 2:**
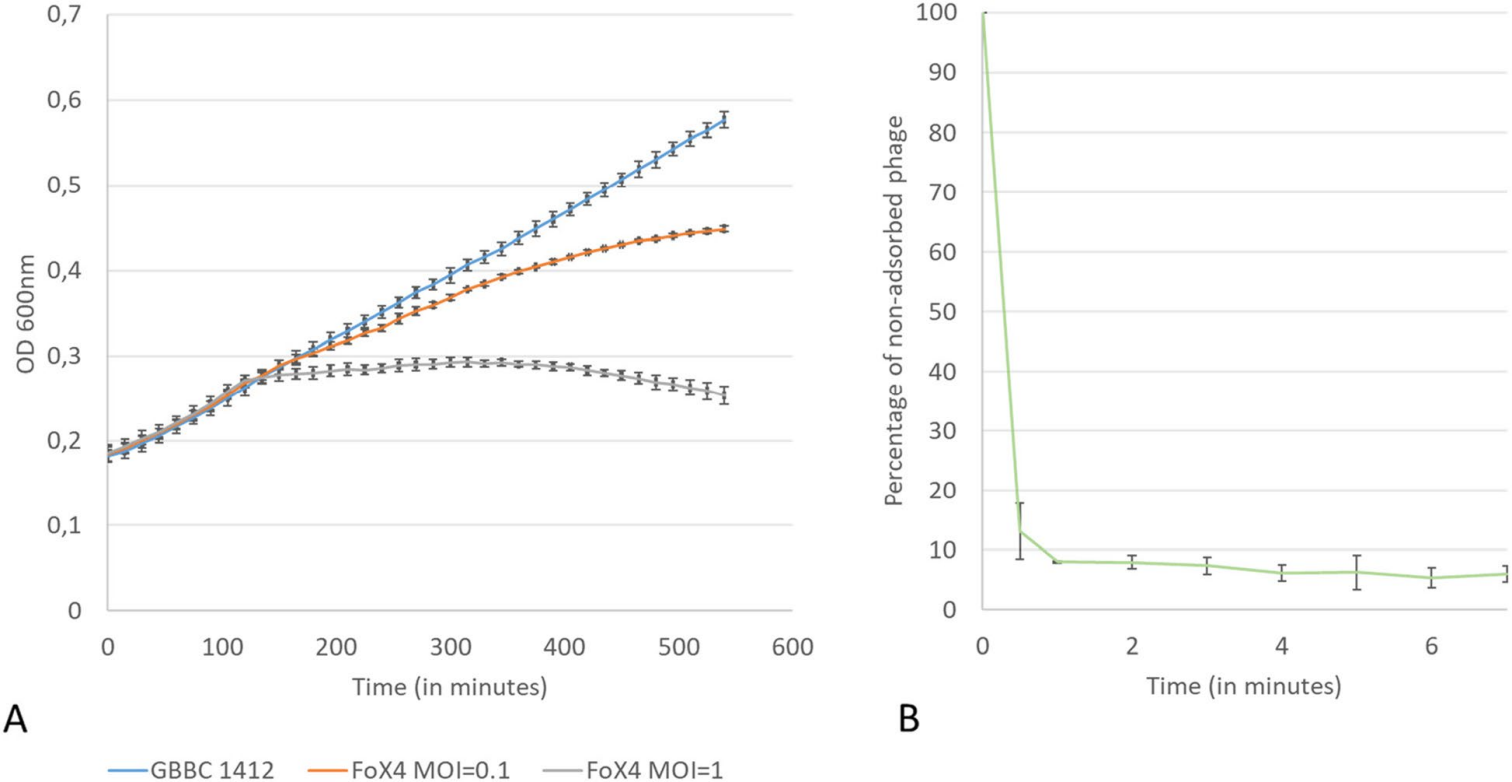
Infection characteristics of phage FoX4. A. Killing curve of FoX4 with two different MOIs (0.1 – 1), for which the optical density at 600 nm was monitored over a time span of 550 min on Xcc strain GBBC 1484. Error bars show the standard deviation. B. Adsorption curve of FoX4 on Xcc strain GBBC 1484 showing the percentage of non-adsorbed phages over a time span of 8 min. Error bars represent the standard deviation

We further investigated the efficacy of FoX4 to lyse a bacterial culture and its adsorption kinetics (Fig. 2). At an MOI of 0.1, FoX4 only displayed a minor influence on the bacterial growth compared to the negative control. When a tenfold higher phage concentration was applied, a reduction of the bacterial growth was observed after 120 min with a clear decrease of the OD after 350 min. Despite its relatively limited lysis activity in an exponentially growing culture, FoX4 adsorbed rather fast to its host. After one minute, 92% of phage particles are adsorbed to the bacterial cells resulting in an adsorption constant of 5.69 × 10^–9^ mL/min.

### FoX4 is an orphan phage of a new phage genus

**Fig. 3:**
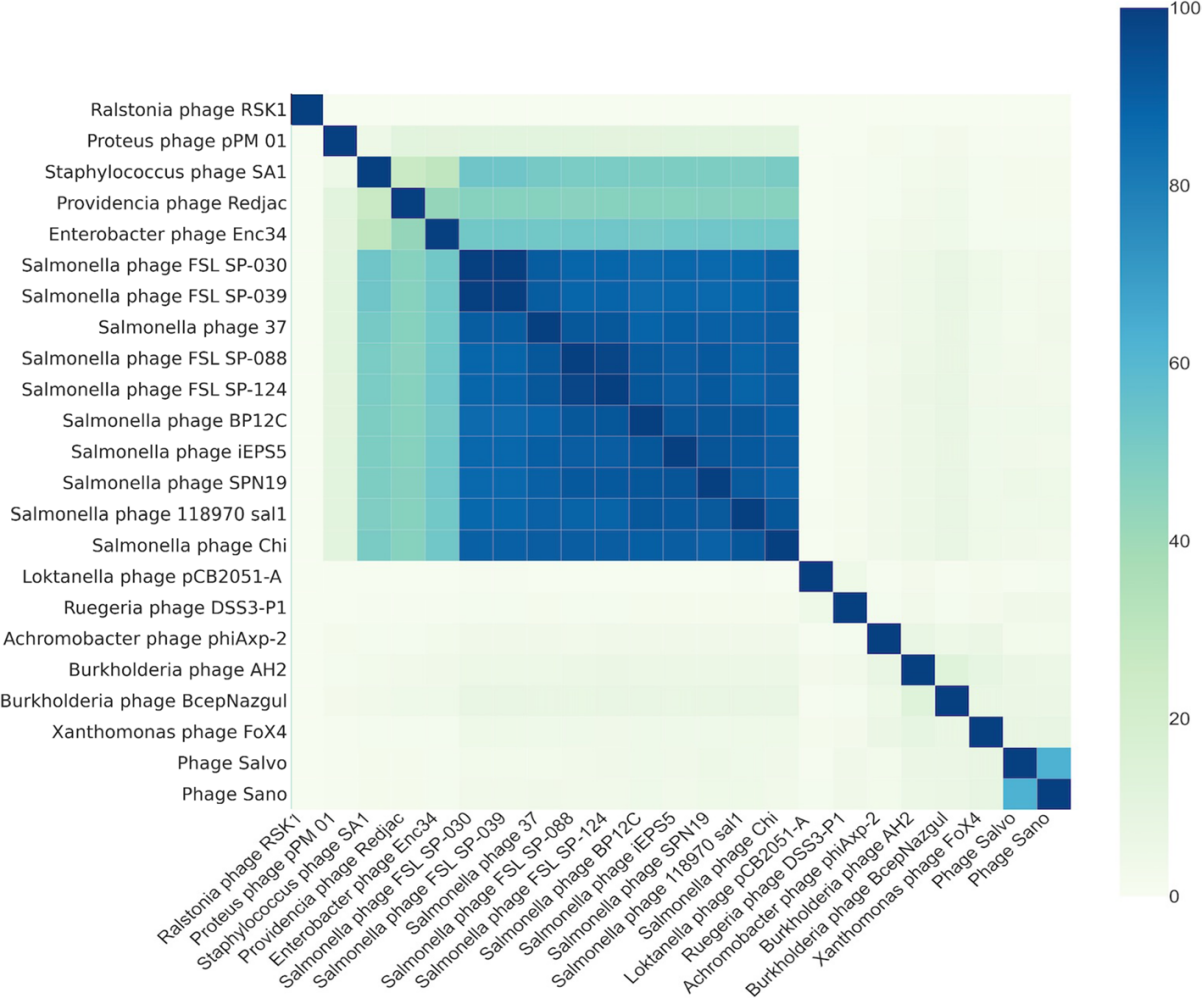
Heatmap of the phage genomes predicted to be in the same subfamily as FoX4 based on a VIRIDIC analysis. Genome similarities above 70% represent one phage genus. The *Foxquatrovirus* genus is most related to the *Sanovirus, Nazgulvirus* and *Ahduovirus* genera. Most dominantly in our analysis is the *Chivirus* genus

Whole genome sequencing and de novo assembly of the genome demonstrated that the phage had a relatively low similarity to other phages in NCBI. Based on a VipTree analysis, its closest neighbors were *Burkholderia* phage BceS AH2 along with *Xylella* phages Sano and Salvo and *Burkholderia* phage BcepNazgul. We ran a GRAViTy analysis on the genome as described by Turner et al. 2021 showing that FoX4 clusters together with other siphoviruses [3] (Supplementary Figure 1). We selected the genomes of phages sharing between 0.05 and 0.1 CGJ (composite generalized Jaccard) distance to investigate the most related genomes (Supplementary Figure 2). Based on this relatedness, we distinguished six groups sharing over 0.15 CGJ within this specific cluster. We further zoomed in on the most related phage genomes to FoX4 (> 0.4 CGJ), which were incorporated in a VIRIDIC analysis (Fig. 3). Here, we could clearly see that within this particular subcluster as determined by the GRAViTy analysis, the percentage identity was relatively low. FoX4 can therefore be considered an orphan phage, distantly related to, but only sharing low sequence identities with, BcepNazgul (*Nazgulvirus* genus), BceS AH2 (*Ahduovirus* genus), Sano and Salvo (*Sanovirus* genus). Within this subcluster though, we could distinguish the *Chivirus* genus as well with main representative *Salmonella* phage chi. Altogether, these data suggest that FoX4 represents a new phage genus *Foxquatrovirus*, which was ratified by the ICTV.

### Genome architecture of FoX4 and confirmation of the structural cassette by mass spectrometry

**Fig. 4:**
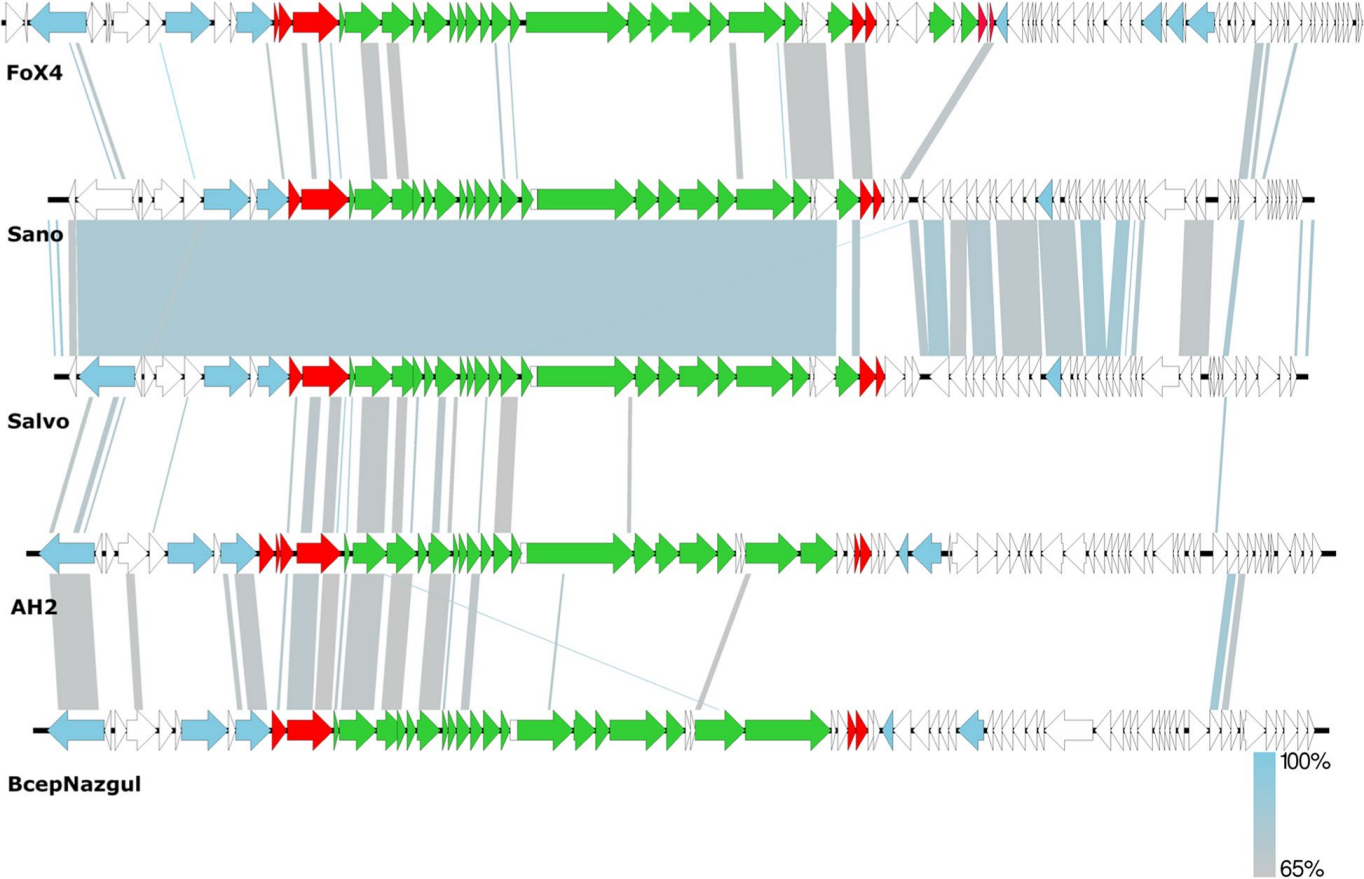
Genome maps of FoX4 and its closest relatives *Xylella* phages Sano and Salvo and *Burkholderia* phages AH2 and BcepNazgul. ORFs associated with replication are given in blue, lysis in red, the structural cassette in green and hypothetical proteins in white. Sequence similarity between the genomes was assessed with BLASTn

When comparing the genomes of FoX4 with its closest relatives, we observed a highly similar genome architecture between the different phages (Fig. 4). Four gene clusters could be distinguished which were shared among all relatives. Notably, the early genes were transcribed on the reverse strand as well as the structural and lysis cassette. Just like its relatives, FoX4 showed potential relicts of a temperate lifestyle encoded in its genome with recombination-related proteins and Cro-like repressors.

Based on mass spectrometry, we determined the full structural cassette of FoX4 (Table 1). As such, we confirmed that gp18-21, gp25-26, gp28-34, gp36, gp43 and gp44, encoded within the putative structural cassette, indeed code for proteins of the FoX4 virion. Six previously hypothetical proteins can now be annotated as part of the structural cassette encoding virion-associated proteins. Interestingly, peptides from gp60 were also found in the sample. As this gene is not embedded in the structural cassette, it is difficult to conclude whether it is truly part of the virion. Maybe more likely, this recombination protein is produced in large quantities during the infection cycle and therefore detected in the mass spectrometry analysis.

**Table 1:** Mass spectrometry analysis of the FoX4 virion. The respective ORF is given along with the number of unique peptides, the percentage of the sequence covered by the peptide and the predicted molecular weight of the protein

Hit	Functional annotation	Unique peptides	Sequence coverage	Mol weight kDa
Gp18	Phage head, portal protein B	9	22.3	60.16
Gp19	Prohead protease ClpP	5	12.5	46.38
Gp20	Decorator protein	5	66.4	14.43
Gp21	Major capsid protein	15	58.5	39.48
Gp25	Hypothetical protein	5	56.8	16.17
Gp26	Hypothetical protein	11	65.1	29.17
Gp28	Tape measure protein	10	8.9	159.72
Gp29	Hypothetical protein	2	9.3	34.63
Gp30	Hypothetical protein	7	27.4	34.86
Gp31	Tail assembly protein	7	15.8	63.22
Gp32	Putative FAD/FMN dehydrogenase	3	13.3	30.70
Gp33	Tail protein	7	14.1	89.10
Gp34	Putative SGNH hydrolase protein	4	27.7	27.58
Gp36	Hypothetical protein	4	24.2	34.70
Gp43	Hypothetical protein	8	83.1	19.55
Gp44	Putative tail fiber protein	5	22	38.96
Gp69c	Recombination-associated protein RdgC	3	8.3	45.32

## Discussion

As the interest in the application of phages for biological control of bacteria increases and the cost of sequencing further decreases, the number of accessible phage genomes will also keep on increasing. This creates challenges yet unique opportunities to fleece out the taxonomical relatedness of bacteriophages based on genome sequence. In this research, we investigated the microbiological and genomic characterization of *Xanthomonas* phage FoX4. In our isolation procedure, we chose to use a soil-based isolation as crop residues and infested soil are believed to function as a reservoir for Xcc [8]. The field sampled for this study was used for crucifer production in consecutive years, creating a unique opportunity to sample infested soil. Similarly, Papaianni and colleagues isolated Xcc phages from the rhizosphere of Kohlrabi [16]. Yet, Xcc phage Carpasina was isolated from the leaves of infected brassica leaves [27], also demonstrating the potential of phage isolations from leaves rather than soil. [27].

Siphovirus FoX4 has a narrow host range infecting only a minor subset of the strains in our collection. Interestingly, the host range of FoX4 is highly similar to Xcc phage FoX6 as previously reported by our group [5]. A more detailed analysis of the receptor of FoX6 showed that this phage most likely recognizes lipopolysaccharides (LPS) on the Xcc cell wall. As FoX4 appears to infect the same strains as FoX6, chances are high that FoX4 and FoX6 require similar phage susceptibility determinants. Yet, there is a difference in the host range between FoX4 and FoX6 as FoX4 can infect historical reference strains while these strains were resistant to FoX6. This example not only demonstrates the intimate interaction between the phage and its bacterial host, but also the need for a detailed host range analysis in order to design phage biocontrol applications. Additionally, in recent years, diverse types of phage resistance mechanisms were reported that interrupt phage infection. We are only beginning to understand the distribution of these mechanisms among bacterial strains and how they impact phage infection [28].

Based on its genome, FoX4 can be considered an orphan phage and representative of a new phage genus [3]. The closest relatives, *Xylella* phages Sano and Salvo, and *Burkholderia* phages AH2 and BcepNazgul are quite distant based on the genome sequence only sharing 23.46, 24.15, 22.24 and 19.64% of Blastn identity, respectively. These phages represent three different phage genera. Despite this low identity, our analysis showed that there is indeed relatedness between the aforenoted phage genera.

## Supplementary Information

**Additional file 1. **

**Additional file 2.**

**Additional file 3.**

## Data Availability

Genome data for FoX4 is accessible on NCBI under the accession number MT161385.1.
