# Supplementary material for: Isolation, characterization and genome analysis of an orphan phage FoX4 of the new Foxquatrovirus genus
Source: BMC Microbiol. 2022 Dec 13;22:304. doi: 10.1186/s12866-022-02719-3 (PMC9746216; doi:10.1186/s12866-022-02719-3)
Supplement: Supplementary file 1 — Additional file 1. [file 12866_2022_2719_MOESM1_ESM.docx]

Supplementary Table 1 Host range analysis of phage FoX4 on an in-house Xcc collection isolated as previously reported (6). For every strain, the acquisition number, bacterial species, plant origin, geographical origin and isolation year are given. Strains that are susceptible to FoX4 are shown in green. BE – Belgium, SA – South Africa, MU – Mauritius, UK – United Kingdom, NL – The Netherlands, TZ – Tanzania, US – United States.

| **Acquisition number** | **Bacterial species** | **Plant origin** | **Geographic origin** | **Year of isolation** | **FoX4 susceptibility** |
| --- | --- | --- | --- | --- | --- |
| GBBC 820 | *Xanthomonas campestris* pv. *campestris* | *B. oleracea* var. *botrytis* | BE | 1999 |  |
| GBBC 821 | *Xanthomonas campestris* pv. *campestris* | *B. oleracea* var. g*emmifera* | BE | 1999 |  |
| GBBC 932 | *Xanthomonas campestris* pv. *campestris* | *B. oleracea* var. g*emmifera* | BE | 2005 |  |
| GBBC 933 | *Xanthomonas campestris* pv. *campestris* | *B. oleracea* var. *gemmifera* | BE | 2005 |  |
| GBBC 1068 | *Xanthomonas campestris* pv. *campestris* | *B. oleracea* var. *botrytis* | BE | 2010 |  |
| GBBC 1069 | *Xanthomonas campestris* pv. *campestris* | *B. oleracea* var. *botrytis* | BE | 2010 |  |
| GBBC 1070 | *Xanthomonas campestris* pv. *campestris* | *B. oleracea* var. *botrytis* | BE | 2010 |  |
| GBBC 1071 | *Xanthomonas campestris* pv. *campestris* | *B. oleracea* var. *botrytis* | BE | 2010 |  |
| GBBC 1072 | *Xanthomonas campestris* pv. *campestris* | *B. oleracea* var. *botrytis* | BE | 2010 |  |
| GBBC 1073 | *Xanthomonas campestris* pv. *campestris* | *B. oleracea* var. *botrytis* | BE | 2010 |  |
| GBBC 1372 | *Xanthomonas campestris* pv. *campestris* | *B. oleracea* var. *botrytis* | BE | 2011 |  |
| GBBC 1373 | *Xanthomonas campestris* pv. *campestris* | *B. oleracea* var. *botrytis* | BE | 2011 |  |
| GBBC 1412 | *Xanthomonas campestris* pv. *campestris* | *B. oleracea* var. *capitata* | BE | 2011 |  |
| GBBC 1413 | *Xanthomonas campestris* pv. *campestris* | *B. oleracea* var. *capitata* | BE | 2011 |  |
| GBBC 1414 | *Xanthomonas campestris* pv. *campestris* | *B. oleracea* var. *capitata* | BE | 2011 |  |
| GBBC 1415 | *Xanthomonas campestris* pv. *campestris* | *B. oleracea* var. *gemmifera* | BE | 2011 |  |
| GBBC 1416 | *Xanthomonas campestris* pv. *campestris* | *B. oleracea* var. *rubra* | BE | 2011 |  |
| GBBC 1417 | *Xanthomonas campestris* pv. *campestris* | *B. oleracea* var. *gemmifera* | BE | 2011 |  |
| GBBC 1419 | *Xanthomonas campestris* pv. *campestris* | *B. oleracea* var. *sabauda* | BE | 2011 |  |
| GBBC 1420 | *Xanthomonas campestris* pv. *campestris* | *B. oleracea* var. *sabauda* | BE | 2011 |  |
| GBBC 1421 | *Xanthomonas campestris* pv. *campestris* | Drain water | BE | 2011 |  |
| GBBC 1429 | *Xanthomonas campestris* pv. *campestris* | *B. oleracea* var. *botrytis* | BE | 2012 |  |
| GBBC 1430 | *Xanthomonas campestris* pv. *campestris* | *B. oleracea* var. *alba* | BE | 2012 |  |
| GBBC 1431 | *Xanthomonas campestris* pv. *campestris* | *B. oleracea* var. *rubra* | BE | 2012 |  |
| GBBC 1432 | *Xanthomonas campestris* pv. *campestris* | *B. oleracea* var. *sabauda* | BE | 2012 |  |
| GBBC 1443 | *Xanthomonas campestris* pv. *campestris* | *B. oleracea* var. *sabauda* | BE | 2012 |  |
| GBBC 1446 | *Xanthomonas campestris* pv. *campestris* | *B. oleracea* var. *alba* | BE | 2012 |  |
| GBBC 1447 | *Xanthomonas campestris* pv. *campestris* | *B. oleracea* var. *rubra* | BE | 2012 |  |
| GBBC 1448 | *Xanthomonas campestris* pv. *campestris* | *B. oleracea* var. *rubra* | BE | 2012 |  |
| GBBC 1449 | *Xanthomonas campestris* pv. *campestris* | *B. oleracea* var. *rubra* | BE | 2012 |  |
| GBBC 1453 | *Xanthomonas campestris* pv. *campestris* | *B. oleracea* var. *gemmifera* | BE | 2012 |  |
| GBBC 1454 | *Xanthomonas campestris* pv. *campestris* | *B. oleracea* var. *rubra* | BE | 2012 |  |
| GBBC 1455 | *Xanthomonas campestris* pv. *campestris* | *B. oleracea* var. *rubra* | BE | 2012 |  |
| GBBC 1456 | *Xanthomonas campestris* pv. *campestris* | *B. oleracea* var. *rubra* | BE | 2012 |  |
| GBBC 1457 | *Xanthomonas campestris* pv. *campestris* | *B. oleracea* var. *rubra* | BE | 2012 |  |
| GBBC 1458 | *Xanthomonas campestris* pv. *campestris* | *B. oleracea* var. *alba* | BE | 2012 |  |
| GBBC 1467 | *Xanthomonas campestris* pv. *campestris* | Drain water | BE | 2012 |  |
| GBBC 1468 | *Xanthomonas campestris* pv. *raphani* | *B. oleracea* var. *botrytis* | BE | 2012 |  |
| GBBC 1469 | *Xanthomonas campestris* pv. *arboricola* | *B. oleracea* var. *botrytis* | BE | 2012 |  |
| GBBC 1470 | *Xanthomonas campestris* pv. *arboricola* | *B. oleracea* var. *botrytis* | BE | 2012 |  |
| GBBC 1484 | *Xanthomonas campestris* pv. *campestris* | *B. oleracea* var. *gemmifera* | BE | 2012 |  |
| GBBC 3215 | *Xanthomonas campestris* pv. *campestris* | *B. oleracea* var. *botrytis* | BE | 2017 |  |
| GBBC 3216 | *Xanthomonas campestris* pv. *campestris* | *B. oleracea* var. *botrytis* | BE | 2017 |  |
| GBBC 3217 | *Xanthomonas campestris* | *E. vesicaria* ssp. *sativa* | BE | 2017 |  |
| GBBC 3218 | *Xanthomonas campestris* pv. *campestris* | *B. oleracea* var. *botrytis* | BE | 2017 |  |
| GBBC 3220 | *Xanthomonas campestris* pv. *campestris* | *B. oleracea* var. *botrytis* | BE | 2017 |  |
| GBBC 3221 | *Xanthomonas campestris* pv. *campestris* | *B. oleracea* var. *botrytis* | BE | 2017 |  |
| GBBC 3225 | *Xanthomonas campestris* pv. *campestris* | *B. oleracea* var. *alba* | BE | 2017 |  |
| GBBC3226 | *Xanthomonas campestris* pv. *campestris* | *B. oleracea* var. *botrytis* | BE | 2017 |  |
| GBBC 3227 | *Xanthomonas campestris* pv. *campestris* | *B. oleracea* var. *botrytis* | BE | 2017 |  |
| GBBC 3228 | *Xanthomonas campestris* pv. *campestris* | *B. oleracea* var. *botrytis* | BE | 2017 |  |
| GBBC 3229 | *Xanthomonas campestris* pv. *campestris* | *B. oleracea* var. *botrytis* | BE | 2017 |  |
| GBBC 3236 | *Xanthomonas campestris* | *E. vesicaria* ssp. *sativa* | BE | 2017 |  |
| K13014 | *Xanthomonas campestris* pv. *campestris* | *B. oleracea* var. *botrytis* | BE | 2013 |  |
| Xcc 316 | *Xanthomonas campestris* pv. *campestris* | *seed* | SA | 2012 |  |
| Xcc 324 | *Xanthomonas campestris* pv. *campestris* | *seed* | SA | 2012 |  |
| Xcc 372 | *Xanthomonas campestris* pv. *campestris* | *seed* | SA | 2012 |  |
| Xcc 373 | *Xanthomonas campestris* pv. *campestris* | *seed* | SA | 2012 |  |
|  |  |  |  |  |  |
| LMG 565 | *Xanthomonas campestris* pv. *campestris* | *B. oleracea* var. *capitata* | MU | 1947 |  |
| LMG 566 | *Xanthomonas campestris* pv. *campestris* | *Brassica napus* | UK | 1941 |  |
| LMG 568T | *Xanthomonas campestris* pv. *campestris* | *B. oleracea* var. *gemmifera* | UK | 1957 |  |
| LMG 570 | *Xanthomonas campestris* pv. *campestris* | *B. oleracea* var. *botrytis* | UK | 1958 |  |
| LMG 575 | *Xanthomonas campestris* | *Cheiranthus cheiri* | UK | 1970 |  |
| LMG 8052 | *Xanthomonas campestris* pv. *campestris* | *B. oleracea* var. *gemmifera* | NL | 1986 |  |
| LMG 535PT | *Xanthomonas campestris* pv. *campestris* | *Iberis* | TZ | 1954 |  |
| CFBP 5824 | *Xanthomonas campestris* pv. *amoraciae* | *Amoracia lapathifolia* | US | 1939 |  |
| LMG 860PT | *Xanthomonas campestris* pv. *raphani* | *Rhaphanus sativus* | US | 1940 |  |
| LMG 547PT | *Xanthomonas campestris* pv. *barbarea* | *Barbarea vulgaris* | US | 1939 |  |
| LMG 7490PT | *Xanthomonas campestris* pv. *incanae* | *Mathiola incana* | US | 1940 |  |
